# Extreme juvenile gigantomastia with bilateral excision of 16.5 kg (27% of body weight) in a 15-year-old adolescent: a case report

**DOI:** 10.1093/jscr/rjag534

**Published:** 2026-06-26

**Authors:** Ismail Hailouma, Soufyane El Kadiri, Mohamed Amine Gazanayi, Doha Arreyouchi, Ayat Allah Oufkir

**Affiliations:** Department of Plastic, Reconstructive and Aesthetic Surgery, Mohammed VI University Hospital, Oujda, Morocco; Faculty of Medicine and Pharmacy, Mohammed First University, Oujda, Morocco; Department of Plastic, Reconstructive and Aesthetic Surgery, Mohammed VI University Hospital, Oujda, Morocco; Faculty of Medicine and Pharmacy, Mohammed First University, Oujda, Morocco; Department of Plastic, Reconstructive and Aesthetic Surgery, Mohammed VI University Hospital, Oujda, Morocco; Faculty of Medicine and Pharmacy, Mohammed First University, Oujda, Morocco; Department of Plastic, Reconstructive and Aesthetic Surgery, Mohammed VI University Hospital, Oujda, Morocco; Faculty of Medicine and Pharmacy, Mohammed First University, Oujda, Morocco; Department of Plastic, Reconstructive and Aesthetic Surgery, Mohammed VI University Hospital, Oujda, Morocco; Faculty of Medicine and Pharmacy, Mohammed First University, Oujda, Morocco

**Keywords:** juvenile gigantomastia, massive breast hypertrophy, breast reduction, Thorek technique, free nipple–areola complex graft

## Abstract

Juvenile gigantomastia is a rare clinical entity associated with major functional and psychological consequences. We report the case of a 15-year-old adolescent with a body mass index of 24.2 kg/m^2^ who presented with extreme bilateral gigantomastia progressing over 2 years. Reduction mammoplasty using the Thorek technique was performed, with a total excision of 16.5 kg (27% of body weight). The procedure was complicated by postoperative hypovolemic shock requiring blood transfusion and intensive care monitoring. Secondary partial necrosis involving both the nipple and areolar components of the nipple–areola complex subsequently occurred and was treated with a full-thickness skin graft harvested from the inguinal region. Despite these complications, the patient achieved satisfactory functional and esthetic outcomes at 8-month follow-up. This case highlights the severity of extreme juvenile gigantomastia and the importance of staged surgical management.

## Introduction

Juvenile gigantomastia is a rare breast development abnormality occurring during puberty, characterized by rapid, often bilateral and symmetrical enlargement. It is generally considered to be present when breast volume exceeds 1500 g per breast, compared with a normal breast weight estimated at around 300 g [1]. It may appear before menarche and can lead to major physical, functional, and psychological repercussions [2]. Diagnosis is clinical, and management requires a multidisciplinary approach.

Its etiology remains unclear, although local hypersensitivity to estrogen is frequently suggested in the absence of systemic hyperestrogenism [3]. Histology usually reveals benign lobular hyperplasia, sometimes associated with stromal fibrosis. Surgical treatment is indicated in severe forms. When ptosis is marked or the sternal notch-to-nipple distance exceeds 35 cm, free nipple–areola complex (NAC) grafting using the Thorek technique is recommended to reduce the risk of vascular compromise [4].

## Clinical case

We report the case of a 15-year-old girl with no significant past medical history, referred to our department by the endocrinology team for progressive bilateral gigantomastia evolving over 2 years. Endocrine assessment revealed no hormonal abnormalities.

The patient presented with chronic low back pain, postural disturbance, limitation of walking, difficulty with daily activities, and marked psychological distress with altered body image and social withdrawal.

**Figure 1:**
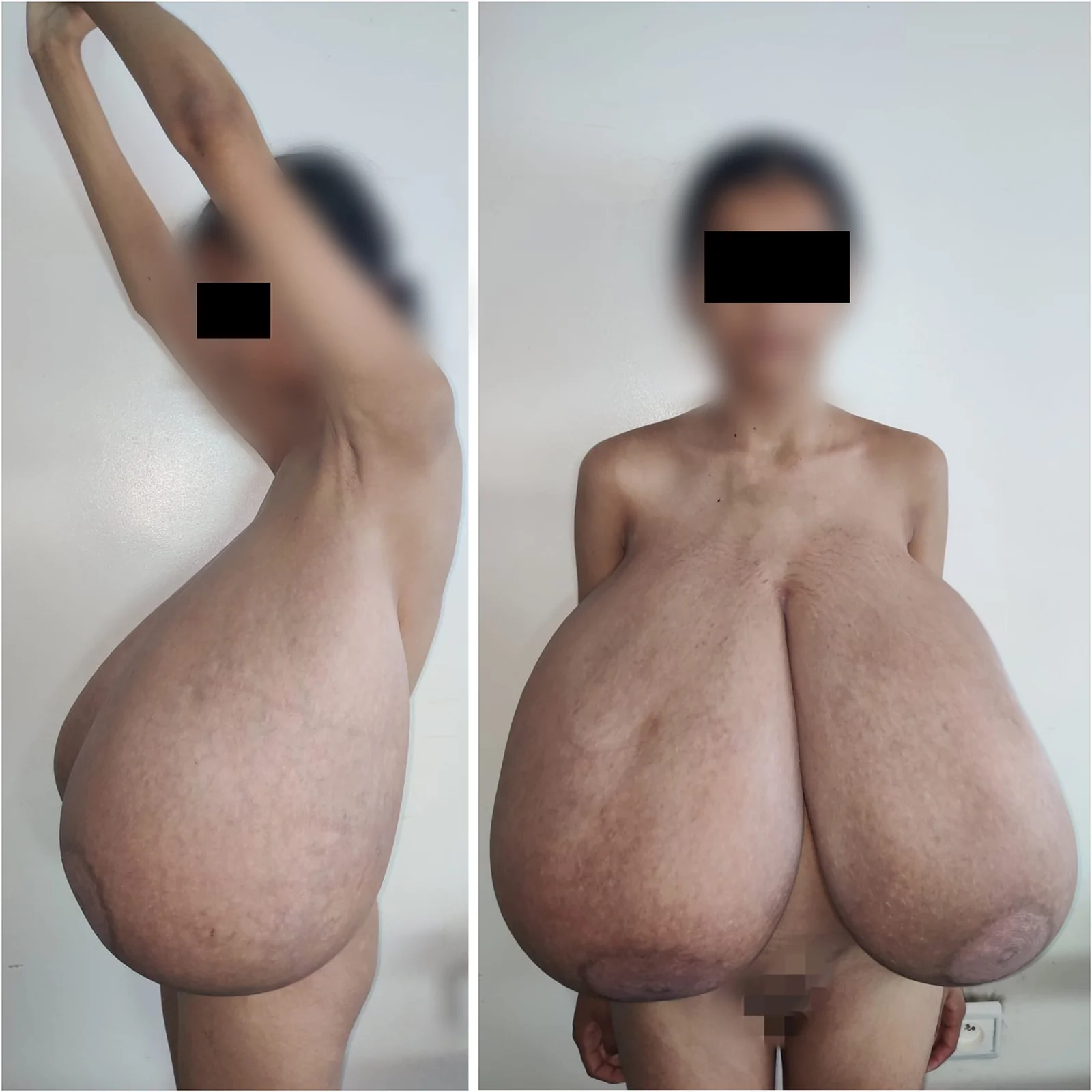
Preoperative appearance of extreme juvenile gigantomastia showing severe bilateral breast hypertrophy and marked ptosis (frontal and lateral views).

On examination, she was 160 cm tall and weighed 62 kg, corresponding to a body mass index of 24.2 kg/m^2^. Massive bilateral gigantomastia with slight asymmetry and severe ptosis was observed. The skin was markedly distended without inflammatory signs. Breast projection measured 39 cm. The sternal notch-to-nipple distance was 46 cm on the right and 47 cm on the left, while areolar diameter measured 12 and 11 cm, respectively. The preoperative appearance is shown in Fig. 1.

**Figure 2:**
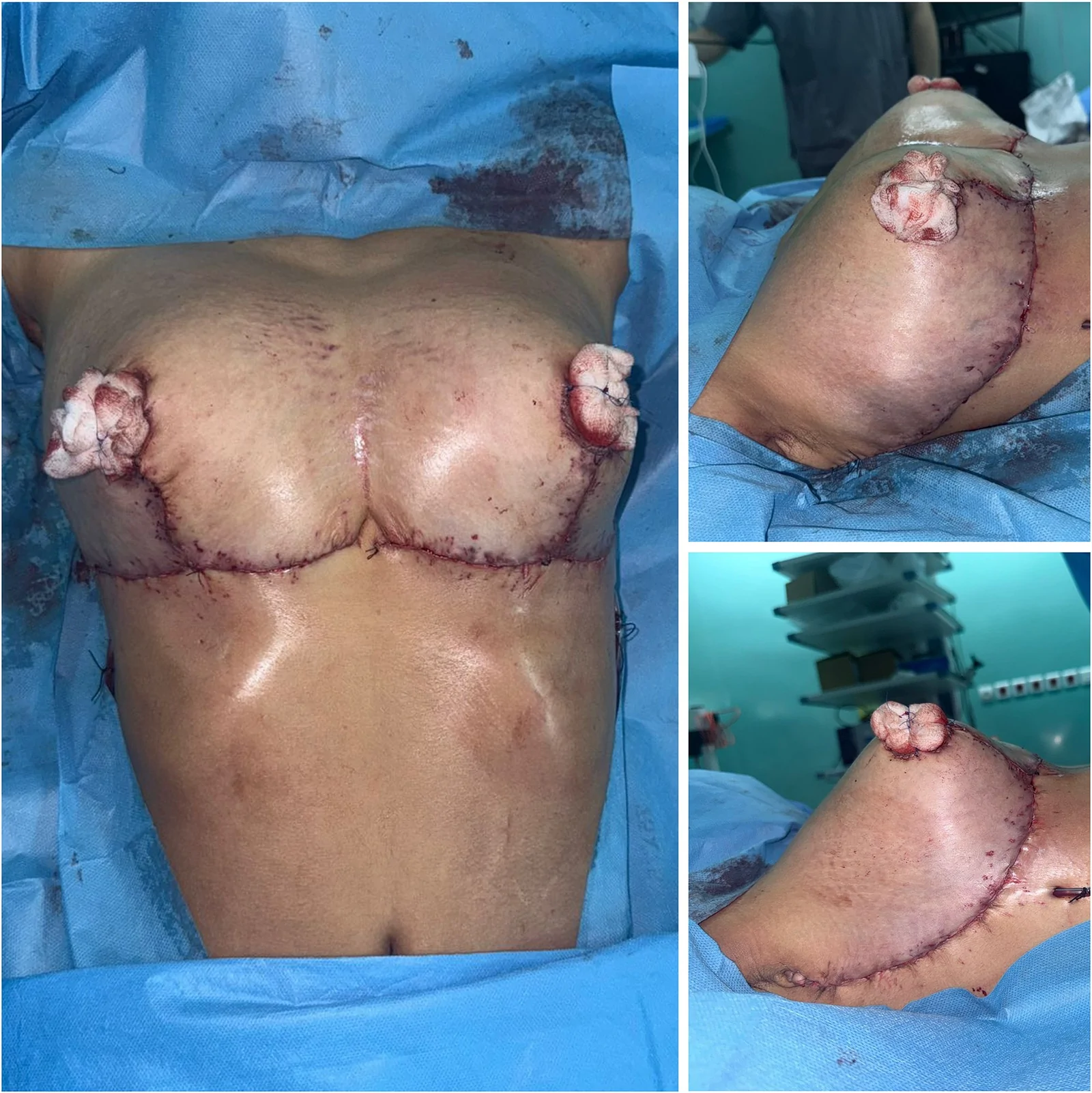
Intraoperative views following reduction mammoplasty using the Thorek technique with free NAC grafting.

Given the severe functional impairment and extreme breast volume, bilateral reduction mammoplasty was indicated. Because of the high risk of pedicle vascular compromise, the Thorek technique with free NAC grafting was selected. Intraoperative views are shown in Fig. 2.

A total of 16.5 kg of breast tissue was resected (8.5 kg from the right breast and 8 kg from the left). The procedure lasted 7 h and was associated with significant blood loss requiring transfusion. Preoperative hemoglobin was 11.8 g/dl and hematocrit was 36.7%. At the end of surgery, hemoglobin decreased to 7.7 g/dl, and the patient developed hypovolemic shock with hemodynamic instability. She was stabilized with aggressive fluid resuscitation and blood transfusion and was monitored in the intensive care unit during the first postoperative night.

The postoperative course was initially favorable. However, partial necrosis involving both the nipple and areolar components of the NAC occurred during follow-up, requiring delayed reconstruction. From the first postoperative week, the patient reported complete resolution of back pain, improved mobility, and marked psychological benefit.

**Figure 3:**
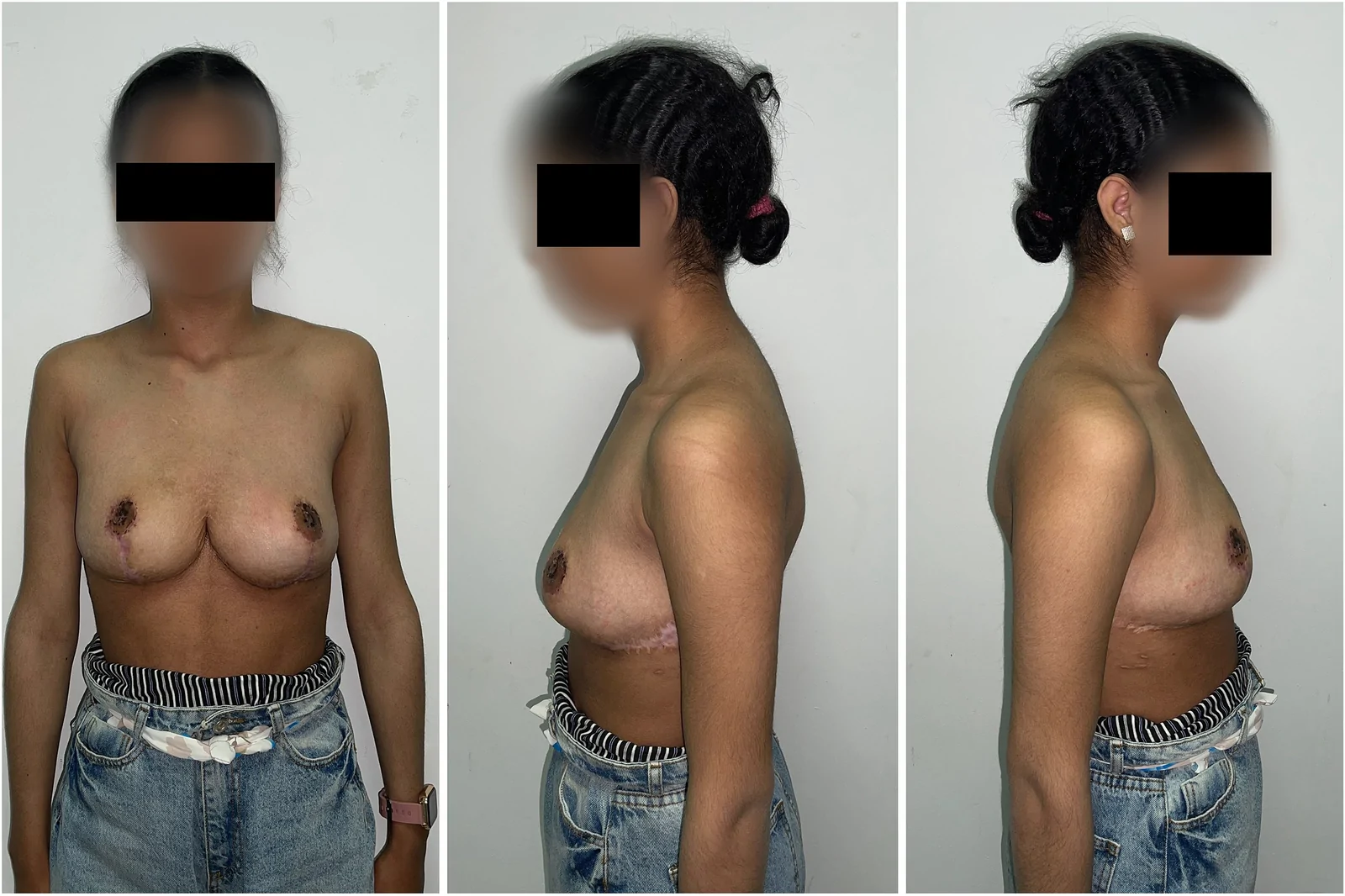
Postoperative appearance at 8-month follow-up showing satisfactory breast contour and stable NAC reconstruction.

Seven months later, secondary NAC reconstruction was performed using a circular full-thickness skin graft harvested from the inguinal region. Complete graft take was achieved with satisfactory pigmentation. The postoperative appearance at 8-month follow-up is shown in Fig. 3.

Histopathological examination of both resected specimens revealed a gynecomastia-like pattern with non-proliferative fibrocystic changes and ductal ectasia, without cellular atypia. Immunohistochemical study showed positive cytokeratin 5/6 (CK5/6) staining, confirming the benign nature of the lesions. No histological signs of malignancy were identified.

## Discussion

Juvenile gigantomastia is a rare condition characterized by excessive and rapid breast growth occurring during puberty, most often without an identifiable cause [5, 6]. Extreme forms are exceptional, particularly when the resected volume exceeds several kilograms. With a total excision of 16.5 kg, our case ranks among the largest reported in an adolescent of this age group.

Several pathophysiological hypotheses have been proposed. The most widely accepted theory suggests hypersensitivity of breast tissue receptors to circulating estrogens in the absence of detectable systemic hormonal abnormality [3]. Anastassiades *et al*. reported absence of estrogen receptors with preserved progesterone receptor expression, suggesting altered local hormonal regulation [3]. A genetic origin has also been considered, particularly in familial and bilateral cases [6].

Histologically, juvenile gigantomastia is generally characterized by epithelial and stromal proliferation without cellular atypia [3, 6, 7]. In our patient, pathology showed a gynecomastia-like pattern with non-proliferative fibrocystic changes and ductal ectasia. Immunohistochemical positivity for CK5/6 further supported the benign nature of the lesions, with no evidence of malignancy.

Clinically, juvenile gigantomastia is associated with severe ptosis, functional limitation, and major psychological distress [8]. In advanced forms, surgery remains the standard treatment, whereas medical therapies such as tamoxifen or gonadotropin-releasing hormone agonists have limited efficacy [6]. When the sternal notch-to-nipple distance exceeds 35 cm or when major reduction is required, the Thorek technique with free NAC grafting is recommended to reduce the risk of pedicle vascular compromise [4, 8]. Modified variations of this technique have also been reported [9].

In our case, the sternal notch-to-nipple distance > 45 cm and the exceptional resection volume justified this approach. Although reliable, the Thorek technique carries a risk of NAC necrosis [6]. Partial necrosis involving both the nipple and areolar components of the NAC occurred and required delayed reconstruction. Secondary reconstruction using a full-thickness inguinal skin graft achieved a satisfactory esthetic result, supporting a staged approach in extreme cases.

Recurrence has been described, particularly with renewed hormonal stimulation, and long-term follow-up is therefore recommended [6]. Psychological support is also important, as emphasized by Aillet *et al*. [10] and Hulard *et al*. [11]. Adjuvant tamoxifen has been proposed to reduce recurrence risk, although evidence remains limited [12]. In our case, no recurrence was observed after 8 months of follow-up.

This report has several limitations. First, it represents a single-patient observation, which limits the generalizability of the findings. Second, the follow-up period of 8 months remains relatively short to assess long-term recurrence or endocrine-related progression.

## Conclusion

Juvenile gigantomastia, although rare, can severely affect the physical and psychosocial well-being of adolescent girls. This case, with bilateral excision of 16.5 kg in a 15-year-old patient, illustrates the extreme severity of the condition. Surgical breast reduction remains the treatment of choice in severe forms. In our case, the Thorek technique allowed safe volume reduction with marked functional and psychological benefit.
