# Correction to: Extreme juvenile gigantomastia with bilateral excision of 16.5 kg (27% of body weight) in a 15-year-old adolescent: a case report

**DOI:** 10.1093/jscr/rjag747

**Published:** 2026-08-21

This is a correction to: Ismail Hailouma, Soufyane El Kadiri, Mohamed Amine Gazanayi, Doha Arreyouchi, Ayat Allah Oufkir, Extreme juvenile gigantomastia with bilateral excision of 16.5 kg (27% of body weight) in a 15-year-old adolescent: a case report, *Journal of Surgical Case Reports*, Volume 2026, Issue 6, June 2026, rjag534, https://doi.org/10.1093/jscr/rjag534

The names of the first author were erroneously transposed and have been corrected in the paper to read: “Ismail Hailouma”.

